# Effects of Dietary Fish Meal Replaced by Fish Steak Meal on Growth Performance, Antioxidant Capacity, Intestinal Health and Microflora, Inflammatory Response, and Protein Metabolism of Large Yellow Croaker *Larimichthys crocea*

**DOI:** 10.1155/2023/2733234

**Published:** 2023-12-20

**Authors:** Dianguang Zhang, Yunzong Zheng, Xuexi Wang, Dejuan Wang, Hongjie Luo, Wenbo Zhu, Weini Zhang, Zhengbang Chen, Jianchun Shao

**Affiliations:** ^1^State Key Laboratory of Mariculture Breeding, Key Laboratory of Marine Biotechnology of Fujian Province, College of Marine Sciences, Fujian Agriculture and Forestry University, Fuzhou 350002, China; ^2^Fuzhou Haima Feed Co. Ltd., Fuzhou 350311, China; ^3^Fuzhou Institute of Oceanography, Fuzhou 350108, China

## Abstract

Although fish steak meal (FSM) is a potentially available protein source, its efficiency as a fish meal (FM) substitute remains unclear to date. To this end, this study was carried out to determine the effects of dietary FM replaced by FSM on growth performance, antioxidant capacity, intestinal health and microflora, inflammatory response, and protein metabolism of large yellow croaker. Five isolipidic and isonitrogenous diets were formulated by substituting FM with FSM at levels of 0% (FSM0, control diet), 25% (FSM25), 50% (FSM50), 75% (FSM75), and 100% (FSM100), and were fed to juvenile large yellow croaker for 8 weeks. Compared with the control diet, the replacement of 25% dietary FM with FSM did not markedly alter the weight gain (WG) and specific growth rate (SGR). When the FM substitution level was over 25%, WG and SGR markedly reduced. The intestinal structure observation found that the FSM75 and FSM100 diets markedly decreased villus height, villus width, and muscle thickness of the anterior intestine. The FSM75 and FSM100 diets significantly decreased enzyme activities of amylase (AMS), lipase (LPS), trypsin, catalase (CAT), and total superoxide dismutase (T-SOD) and the total antioxidant capacity (T-AOC), and increased the malondialdehyde (MDA) content in the liver of large yellow croaker. The mRNA expression levels of intestinal barrier and inflammatory response-related genes suggested that the FSM50, FSM75, and FSM100 diets significantly decreased the mRNA abundances of intestinal barrier-related genes and anti-inflammatory response-related genes, and increased the mRNA abundances of proinflammatory gene *il-6* in the anterior intestine. The compositions of intestinal microflora displayed that the FSM50, FSM75, and FSM100 diets decreased relative abundances of Firmicutes phylum and increased relative abundances of Proteobacteria phylum. In addition, the results of protein expression levels showed that the phosphorylation level of mammalian target of rapamycin (mTOR) and 4E-binding protein 1 (4E-BP1) in FSM75 and FSM100 groups were markedly reduced. In conclusion, FSM can replace up to 25% dietary FM without compromising the growth performance, intestinal health, and protein metabolism of the large yellow croaker.

## 1. Introduction

It is well-known that fish meal (FM), an excellent protein source, is largely utilized in aquatic feeds to meet the protein requirement of aquaculture animals [1]. However, due to the speedy development of aquaculture industry, there is a huge imbalance in FM between rising demand and short supply [2]. Therefore, it is of utmost importance to seek alternative protein sources to FM for maintaining the healthy and sustainable development of aquaculture industry. Although great progress has been made in the exploration of alternative protein sources for FM substitution in recent years [3–5], currently the pursuit of potential novel protein sources remains a major issue and imperative.

Fish steak meal (FSM), a fish processing byproduct, is consisted of fish bones, guts, head, tail, skin, eyes, scales, and fins. The FSM contains approximately 54.0% crude protein, 15.0% crude lipid, as well as 25.0% ash on a dry matter basis, respectively. Additionally, similar to FM, the FSM possesses some superior properties such as abundant polyunsaturated fatty acids, good feeding attraction activities, and a certain amount of unknown growth-promoting factors, which are not found in other plant and animal protein sources. Notably, since bone and meat meal, and soybean meal are well used in poultry and aquaculture feed industry, FSM has not attracted enough attention in past 20 years, thereby hindering further development of FSM as a potential protein source for FM replacement. To determine the potential of FSM as an aquafeed protein source, several feeding trials have been performed on some fish species, including Atlantic cod, rainbow trout, olive flounder [6–8]. However, to our knowledge, the influences of FM replaced by FSM on large yellow croaker *Larimichthys crocea* remain largely unknown.

In the cause of comprehensively understanding influences of FM replaced by other protein sources on aquaculture animals, various indicators are incorporated into the evaluation system, e.g., growth performance and antioxidant capacity, intestinal health and microflora, muscle composition, and quality, and so on [4, 9]. There is no doubt that the growth performance of fish is one of the most direct indexes to evaluate the quality of aquaculture feed. Body redox homeostasis is required for normal growth and development, prevention of metabolic and immune diseases [10–12]. As one of the important organs of aquatic animals, intestinal tract not only plays crucial roles in the digestion and absorption of nutrients, but also resistance to the invasion of pathogenic microorganisms [13, 14]. Moreover, most researches reveal that the fluctuation of intestinal microflora is closely linked to the health of organisms [15]. Hence, maintaining body redox homeostasis, intestinal health, and microflora balance set up a strong foundation for promoting the healthy growth of aquatic animals. With the improvement of people's living standards, the muscle quality of aquaculture animals has been the main concern of popular consumers and investigators [16], indicating the importance of muscle quality improvement in high-quality development of aquaculture. Collectively, taking these indicators into account can give us an extensive knowledge of potential protein resources, which will help potential protein sources to be utilized more appropriately.

Large yellow croaker is one of the most important mariculture fish in China. Its harvest in China is as high as 254, 224-metric tons in 2021 [17], thus leading to increased demand for high-quality protein sources such as FM as well as soybean meal. Notably, at present, the aquatic feed industry faces two prominent problems: shortage of high-quality protein raw materials and their high price. Consequently, continuing to develop and utilize the potential protein sources remains one of the most effective ways to tackle current challenges. Thus, the present study was performed on large yellow croaker and explored the effects of FM replaced by FSM on growth performance, antioxidant capacity, intestinal health and microflora, inflammatory response, and protein metabolism. It might provide some primary data for utilization of FSM in artificial diets for large yellow croaker.

## 2. Materials and Methods

### 2.1. Experimental Diets and Animal Feeding

Five isolipidic (crude lipid, 9.5%) and isonitrogenous (crude protein, 44.0%) diets were formulated by substituting FM by FSM at levels of 0% (FSM0, control diet), 25% (FSM25), 50% (FSM50), 75% (FSM75), and 100% (FSM100) (Table 1). The FSM and FM were provided by the Fuzhou Haima Feed Co. Ltd., (Fuzhou, China), and the nutritional compositions of FSM and FM were shown in Table 2. First, the ingredients are crushed by a pulverizer and passed through a 100-mesh sieve. Then, the ingredients other than fat sources were added in turn and thoroughly mixed according to the principle from small to large. At last, fish oil, soybean oil, soybean lecithin, as well as water were added and the mixture was stirred well for pelleting. The diameter of diet is 2 mm. The dried diets were sealed with plastic bag, and stored at −20°C.

The juvenile large yellow croaker was obtained from a fishery company in Ningde (Fujian, China), and then acclimated for 2 weeks in floating cages (4.0 m × 2.0 m × 2.5 m, length × width × depth). After the acclimatation, 1,200 juvenile large yellow croaker (19.50 ± 1.75 g) were randomly assigned into 20 floating cages (1.0 m × 1.0 m × 1.5 m, length × width × depth), and each group has four floating cages (60 fish per floating cages). Fish were hand-fed to apparent satiation twice daily (5:30 and 17:30) for 8 weeks. During feeding trial, water temperature, dissolved oxygen level, as well as ammonia nitrogen level were 28.0 ± 0.6°C, 6.5 ± 0.5 mg L^−1^, and 0.15 ± 0.06 mg L^−1^, respectively. The photoperiod is 12 hr light and 12 hr dark in floating cages.

### 2.2. Animals Sampling

After 56 days of feeding, all the fish were fasted for 24 hr and were anesthetized with 100 mg/L MS-222 for sampling and acquiring the weight and number of fish. For intestinal histological observation, the anterior intestines from three fish per cage were scissored on ice and then fixed in 4% paraformaldehyde solution. For monitoring the changes of intestinal microflora, the anterior intestines (mixed) from five fish per cage were isolated and collected into a sterile frozen storage tube for 16S rDNA sequencing. Meanwhile, the dorsal muscles (mixed) were also sampled for determining the protein expression of protein metabolism-related genes. Additionally, the livers (mixed) from the other five fish per cage were sampled for determining the activity of digestive enzymes, the oxidative stress-related parameters, and the anterior intestines (mixed) were sampled for determining the mRNA abundances of inflammatory response-related genes. All the samples except for histological observation both were stored at −80°C.

### 2.3. Analysis of Proximate Composition and Amino Acids Profile

The method for proximate compositions analysis of FSM, FM, and diets, and amino acid profiles analysis of FSM and FM were based on the previous study by Xu et al. [18]. The moisture was measured by drying the sample to a constant weight in an oven at 105°C. The ash content was determined using the burning method at 550°C in a muffle furnace. The crude protein content was measured using the Kjeldahl nitrogen method (N × 6.25) (Kjeltec 2200, FOSS, Denmark). The Soxhlet method (Soxhlet extraction system B-811) by petroleum ether extraction was used to determine crude lipid content. The FSM and FM were freeze-dried, and 30 mg of each sample was hydrolyzed using 15 mL 6 N HCl solution at 110°C for 24 hr. Amino acid profile was determined by the automatic amino acid analyzer (L-8900, Hitachi, Japan).

### 2.4. Intestinal Histological Observation

The preparation of tissue section and staining was based on the previous study by Zhang et al. [19]. The image acquisition of intestinal tissue structure was realized by a microscope (CX31RTSF, Olympus, Japan). The ImagePro Plus6.0 software was adopted to determine muscle thickness (MT), villus height (VH), as well as villus width (VW).

### 2.5. Assays of Hepatic Digestive Enzymes Activity and Oxidative Stress-Related Parameters

The detailed procedures for assays of digestive enzymes activity and oxidative stress-related parameters were according to previous study by Yao et al. [20]. The 0.1-g livers samples were homogenized in 0.9-mL ice-cold buffer (2-mM EDTA, 0.25-M sucrose, 0.02-M Tris-HCl, 0.5-mM phenyl methyl sulphonyl fluoride, 0.1-M sodium fluoride, and 0.01-M *β*-mercapto-ethanol, pH 7.4). They were then centrifuged at 2,500 *g* at 4°C for 10 min. The supernatant was used for the following analysis. The activities of AMS, and LPS were measured at 660 nm and at 420 nm, respectively, using AMS and LPS assay kits (C016-1-1, A054-1-1; Nanjing Jiancheng Bioengineering Institute; Nanjing, China). The trypsin activities were measured at 253 nm using the Trypsin Assay Kit (A080-2; Nanjing Jiancheng Bioengineering Institute; Nanjing, China). The CAT and total superoxide dismutase (T-SOD) activities were measured at 405 nm and at 550 nm, respectively, using CAT and T-SOD assay kits (A007-1-1, A001-1-1; Nanjing Jiancheng Bioengineering Institute; Nanjing, China). The T-AOC was determined at 405 nm with T-AOC assay kit (S0121; Beyotime Biotechnology, Shanghai, China). The MDA contents were determined using the Lipid Peroxidation MDA Assay Kit (S0131S; Beyotime Institute of Biotechnology; Shanghai, China).

### 2.6. Quantitative Real-Time PCR

The detailed procedures of total RNA extraction and cDNA synthesis for anterior intestine were based on the previous study by Zhang et al. [21]. The detailed information on primers were shown in Table 3. The real-time quantitative PCR program was 95°C for 5 min, followed by 40 cycles of 95°C for 10 s, 58°C for 10 s, and 72°C for 20 s. The 2^−*ΔΔ*Ct^ method was utilized to determine fold-change of their gene expression to control (FSM0) group.

### 2.7. Gut Microflora

The DNA sequencing was completed by Majorbio Bio-Pharm Technology Co. Ltd. (Shanghai, China). The detailed experimental process was shown by Wang et al. [22]. After sequencing work was completed, the alpha-diversity, bacterial communities, and linear discriminate analysis (LDA) both were well accomplished by the bioinformatics analysis cloud platform of Majorbio Bio-Pharm Technology Co. Ltd. (Shanghai, China).

### 2.8. Western Blot

The phosphorylation levels of mTOR and 4-EBP were measured to determine influences of FM replaced by FSM on protein metabolism in the muscle of large yellow croaker. The detailed procedures were according to the previous study by Chen et al. [23]. The primary antibodies are anti-rabbit mTOR (1 : 1000, #2972, Cell Signaling Technology; MA, USA), anti-rabbit phospho-mTOR (Ser2448) (1 : 1000, #5536, Cell Signaling Technology; MA, USA), anti-rabbit 4E-BP1 (1 : 1000, #9452, Cell Signaling Technology; MA, USA), anti-rabbit phospho-4E-BP1 (Thr37/46) (1 : 1000, #2855, Cell Signaling Technology; MA, USA), anti-mouse *β*-actin (1 : 5000, AC004, ABclonal Technology; Wuhan, China), as well as anti-rabbit *β*-tubulin (1 : 5000, AC008, ABclonal Technology; Wuhan, China), respectively. The secondary antibodies are HRP-conjugated anti-rabbit IgG antibody (1 : 10000, 7074, Cell Signaling Technology; MA, USA) and HRP-conjugated mouse anti-rabbit IgG antibody (1 : 10000, 5127, Cell Signaling Technology; MA, USA). Quantification of protein bands was accomplished by the ImageJ software.

### 2.9. Statistical Analysis

The SPSS 19.0 software was used to perform the statistical analysis. Results were shown as the mean ± SD (standard deviation). First, results were tested for normality and homogeneity of variances. Then, results were analyzed by the polynomial contrasts to determine the pattern of linear and quadratic. At last, one-way ANOVA and Duncans multiple range test were used to analyze the differences among these results. The significance threshold was *P* < 0.05.

## 3. Results

### 3.1. Growth Performance and Feed Utilization

Results showed that replacing FM with FSM did not markedly alter the survival rate (Table 4). Compared with control group, the replacement of 25% FM with FSM did not markedly alter the WG, SGR, feed conversion rate (FCR), as well as protein efficiency ratio (PER) (Table 4). When the FM substitution level was higher than 25%, WG, SGR, and PER markedly reduced, and FCR markedly increased (Table 4).

### 3.2. Digestive Enzymes Activity and Antioxidant Parameters

For digestive enzymes activity, compared with control diet, the AMS, LPS, and trypsin activities markedly reduced when the substitution level of FM by FSM was over 25% (Table 5). For antioxidant parameters, CAT activities in FSM75 and FSM100 groups were obviously lower than those in the control group (Table 5). Compared with control group, T-SOD activities in replacement groups significantly decreased (Table 5). When replacement level of FM by FSM was over 25%, T-AOC markedly reduced compared with the control diet (Table 5). MDA contents of FSM75 and FSM100 groups were markedly higher than those in the control group (Table 5). There were obvious negative linear and quadratic trends between the replacement level of FM with FSM and the enzyme activities of AMS, LPS, trypsin, CAT, T-SOD, and T-AOC. On the contrary, there were obvious positive linear and quadratic trends between the substitution level of FM with FSM and the MDA content.

### 3.3. Intestinal Morphology

**Figure 1:**
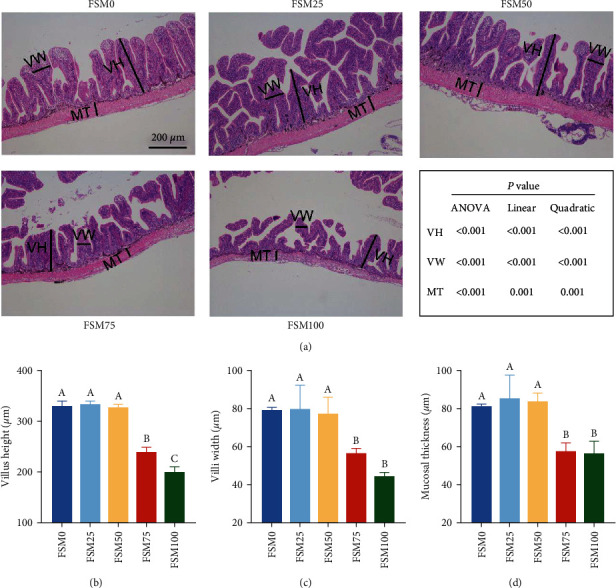
The effects of fish meal replaced by fish steak meal on anterior structure of large yellow croaker. (a) Representative image of anterior intestine histochemistry (stained by haematoxylin and eosin). The scale bar = 200 *μ*m. (b) Villus height. (c) Villus width. (d) Mucosal thickness. FSM, fish steak meal; MT, mucosal thickness; VH, villus height; and VW, villus width. Values are shown as mean ± SD (*n* = 3). Letters (A–C) denote significance *P* < 0.05.

Compared with control diet, FSM25 and FSM50 diets did not markedly alter the VH, VW, and MT of anterior intestine. Compared with control group, anterior intestinal VH, VW, and MT significantly decreased in FSM75 and FSM100 groups (Figure 1). There were significant negative linear and quadratic trends between the substitution level of FM with FSM and the VH, VW, and MT.

### 3.4. Relative mRNA Expression Levels of Intestinal Barrier and Inflammatory Response-Related Genes in the Anterior Intestine of Large Yellow Croaker

**Figure 2:**
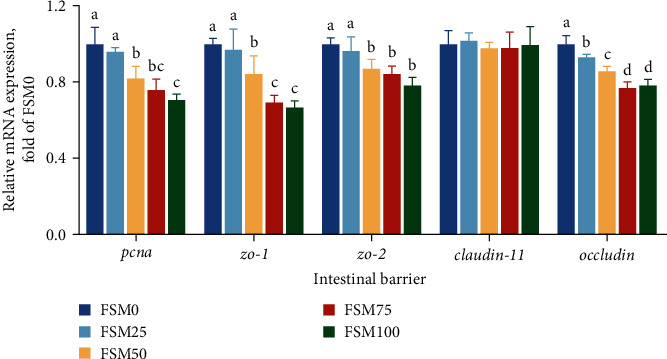
The effects of fish meal replaced by fish steak meal on mRNA expression levels of intestinal barrier-related genes in the anterior intestine of large yellow croaker. FSM, fish steak meal; *pcna*, proliferating cell nuclear antigen; *zo-1*, zonula occludens-1; and *zo-2*, zonula occludens-2. Values are shown as mean ± SD (*n* = 4). Letters (a–d) denote significance *P* < 0.05.

Compared with control group, FSM50, FSM75, as well as FSM100 diets markedly reduced mRNA abundances of proliferating cell nuclear antigen (*pcna*), zonula occludens-1 (*zo-1*), as well as *zo-2*, but FSM25 diet did not markedly alter mRNA abundances of *pcna*, *zo-1*, as well as *zo-2* (Figure 2). There was no significant difference in mRNA abundances of *claudin-11* among five groups (Figure 2). Compared with control group, replaced groups remarkedly reduced mRNA abundance of *occluding* (Figure 2).

**Figure 3:**
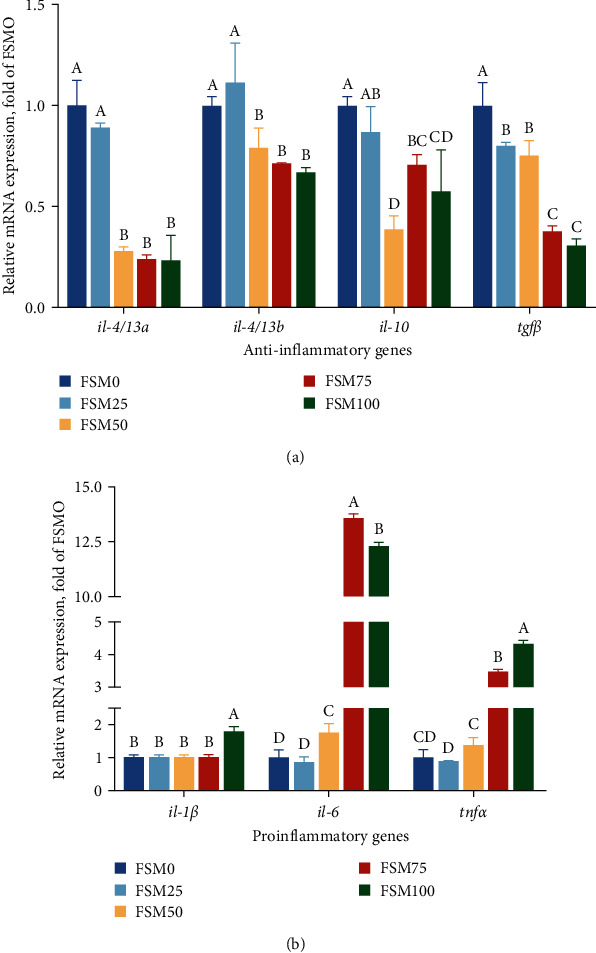
The effects of fish meal replaced by fish steak meal on mRNA expression levels of inflammatory response-related genes in the anterior intestine of large yellow croaker. (a) The relative mRNA expression levels of anti-inflammatory genes. (b) The relative mRNA expression levels of proinflammatory genes. FSM, fish steak meal; *il-10*, interleukin-10; *il-1β*, interleukin-1*β*; *il4-13a*, interleuk*in-4-13 a; il4-13b*, interleukin-4-13b; *il-6*, interleukin-6; *tgfβ*, transforming growth factor *β*; and *tnfα*, tumor necrosis factor *α*. Values are shown as mean ± SD (*n* = 4). Letters (A–D) denote significance *P* < 0.05.

Compared with control group, the mRNA abundances of interleukin-4/13a (*il-4/13a*), *il-4/13b*, and *il-10* significantly decreased when the substitution level of FM by FSM was over 25% (Figure 3(a)). Compared with control group, the mRNA levels of transforming growth factor *β* (*tgfβ*) significantly decreased in the other groups (Figure 3(a)). Compared with control diet, FSM100 diet markedly increased mRNA levels of *il-1β*, and FSM50, FSM75, and FSM100 diets significantly decreased mRNA abundance of *il-6* (Figure 3(b)). Compared with control group, the mRNA levels of tumor necrosis factor *α* (*tnfα*) markedly reduced in FSM75 and FSM100 groups (Figure 3(b)).

### 3.5. The Influences of FM Replaced by FSM on the Intestinal Microflora of Large Yellow Croaker

**Figure 4:**
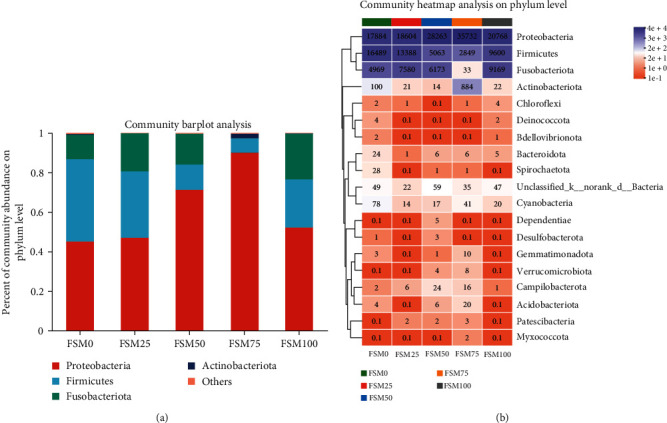
The effects of fish meal replaced by fish steak meal on intestinal microbial community structure of large yellow croaker. (a) The percent of community abundance on phylum level. (b) The community heatmap analysis on phylum level. FSM, fish steak meal.

**Figure 5:**
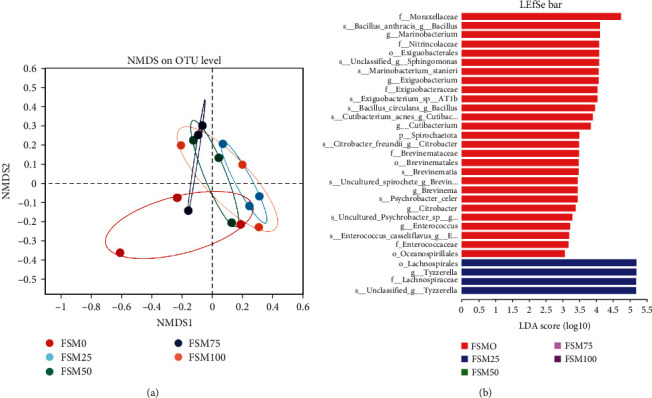
The effects of fish meal replaced by fish steak meal on intestinal microflora of large yellow croaker. (a) The analysis of nonmetric multidimensional scaling (NMDS) on OTU level. (b) Histogram of linear discriminant analysis (LDA) scores for differentially abundant taxon. FSM, fish steak meal.

In general, 328 operational taxonomic units (OTUs) were identified and assigned to 19 phyla, 35 classes, 87 orders, 142 families, 217 genera, and 262 species. Results of alpha diversity for intestinal microflora showed that compared with control group, replaced group did not significantly alter alpha diversity indexes such as sobs, shannon, simpson, ace, chao, and coverage (Table 6). But there is an obvious downward trend for ace and chao when substitution level of FM by FSM was over 25% (Table 6). Furthermore, the analysis of intestinal microbial community structure suggested that there were 59, 16, 19, 48, and 51 unique OTUs for FSM0, FSM25, FSM50, FSM75, and FSM100 groups, respectively (Figure S1). In addition, the results exhibited that Fusobacteriota, Firmicutes, as well as Proteobacteria were major bacterial phylum (Figure 4(a); Figure S2). Notably, compared with control group, relative abundances of Proteobacteria phylum were increased and relative abundances of Firmicutes phylum were decreased when the substitution level of FM by FSM was over 25% (Figures 4(a) and 4(b)). The analysis of nonmetric multidimensional scaling (NMDS) revealed distinct clustering between microbiota from fish fed FSM0, FSM25, FSM50, FSM75, and FSM100 diets (Figure 5(a)). The LEfSe analysis was conducted to identify the difference in intestinal microbial community composition of large yellow croaker fed FSM0, FSM25, FSM50, FSM75, and FSM100 diets. The results indicated that compared with control diet, replaced diets markedly reduced relative abundance of family Moraxellaceae, genus Bacillus, genus Marinobacterium, family Nitrincolaceae, order Exiguobacterales, and class Brevinematia (Figure S3; Figure 5(b)).

### 3.6. The Influences of FM Replaced by FSM on Phosphorylation Level of mTOR (Ser2448) and 4E-BP1 (Thr37/46) in the Dorsal Muscle of Large Yellow Croaker

**Figure 6:**
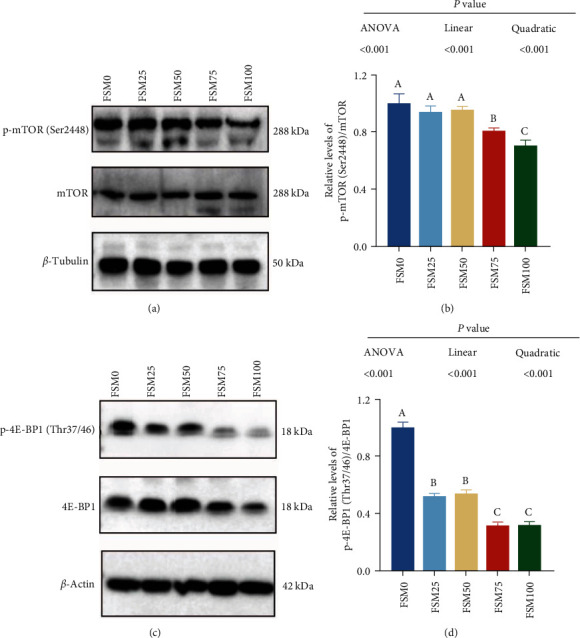
The effects of fish meal replaced by fish steak meal on phosphorylation level of mTOR (Ser2448) and 4E-BP1 (Thr37/46) in the dorsal muscle of large yellow croaker. (a, b) The phosphorylation level of mTOR (Ser2448). (c, d) The phosphorylation level of 4E-BP1 (Thr37/46). FSM, fish steak meal; mTOR, mammalian target of rapamycin; p-mTOR, phosphorylated mammalian target of rapamycin; 4E-BP1, eukaryotic translation initiation factor 4E-binding protein 1; p-4E-BP1, phosphorylated eukaryotic translation initiation factor 4E-binding protein 1.

Compared with control group, FSM75 and FSM100 diets markedly reduced phosphorylation level of mTOR (Ser2448) in the dorsal muscle of large yellow croaker (Figures 6(a) and 6(b)). Compared with control group, the phosphorylation level of 4E-BP1 (Thr37/46) in FSM25, FSM50, FSM75, and FSM100 groups were markedly decreased (Figures 6(c) and 6(d)). There were significant negative linear and quadratic trends between the substitution level of FM with FSM and the phosphorylation level of mTOR (Ser2448) and 4E-BP1 (Thr37/46).

## 4. Discussion

In this study, we found that substituting dietary FM by FSM made no significant difference on survival rate of large yellow croaker. Consistently, similar results were also found in juvenile Japanese flounder [8, 24]. In Atlantic cod, Toppe et al. [6] reported that fish bone meal (55.8% crude protein; 7.7% crude lipid), called FSM, could replace up to 51% dietary FM and its replacement did not significantly affect the WG, SGR, FCR, and PER. In Japanese flounder, Uyan et al. [24] pointed out that FSM (79.9% crude protein; 14.1% crude lipid), prepared from the tuna muscle byproduct, could replace 50% dietary FM without a decrease of growth performance and FCR. In this study, our results suggested that without altering growth performance and feed utilization, the FSM (54.15% crude protein; 14.56% crude lipid) was capable of substituting 25% dietary FM for large yellow croaker. Notably, the quality of FSM from different sources (fish species and constituent parts) varies greatly. In addition to this, previous studies have illustrated that the tolerance abilities of aquatic animals to the same available protein sources are closely associated with the species and growth stage of tested aquatic animals [25]. Hence, it is not difficult for us to understand the difference in the proportion of FSM replaced for dietary FM among the different aquatic animals.

Apart from the growth performance and feed utilization, the digestive enzymes activity was also an important index for determining the potential of available protein sources as dietary FM substitutes for aquatic animals [26]. The AMS, LPS, and trypsin, three major digestive enzymes, are essential for the initial degradation of dietary starch, lipid, and protein, respectively [26]. The higher digestive enzymes activity represents the stronger digestive capacity. In this study, while the FM substitution level was over 25%, the AMS, LPS, and trypsin enzymes activity were significantly decreased. From these findings, we could conclude that high-dietary FSM inclusion level would dramatically undercut the digestive capacity for large yellow croaker. Multiple works have confirmed that increment of digestive enzymes activity can enhance feed utilization to a large extent and then improve growth performance [27]. Consequently, we speculated that the high-dietary FSM supplementation-induced decrease of digestive enzymes activity might account for the reduction of growth performance and feed utilization in FSM50, FSM75, as well as FSM100 groups.

The body's antioxidant capacity mirrors the organism's capability to scavenge external or internal stimuli-induced overproduction of reactive oxygen species (ROS) [28]. The reduction of antioxidant capacity will result in the redundancy of ROS as well as oxidative stress, and further evoke oxidative stress induced-lipid peroxidation and DNA damage [29]. The body's antioxidant capacity and oxidative stress status are characterized by some antioxidant enzymes and lipid peroxidation products such as CAT, SOD, T-AOC, and MDA, etc. The SOD, the first antioxidant enzyme involved in counteracting oxidative stress, catalyzes O_2_^−^. to oxygen and H_2_O_2_ [30]. The CAT then converts H_2_O_2_ into water through a catalytic reaction [30]. The T-AOC is the comprehensive embodiment of body's total antioxidant capacity [27]. In this study, compared with control diet, FSM75 and FSM100 diets significantly decreased CAT activity, and replaced diets significantly decreased T-SOD activity in the liver of large yellow croaker. Besides, when FM substitution level was over 25%, the T-AOC remarkably decreased. MDA is a signature product of oxidative stress and the increment of MDA content implies an increase in oxidative stress damage [23]. On the contrary to the trend of antioxidant capacity, the MDA content significantly increased when the FM substitution level was over 25%. Taken together, these results suggested that inappropriate dietary FSM addition would impair hepatic antioxidant capacity and increase risk of hepatic oxidative stress damage for large yellow croaker. Additionally, a large amount-number of studies have illustrated that resisting oxidative stress injury and maintaining redox homeostasis is required for healthy growth of aquatic animals [27]. Therefore, in the context of exploring the feasibility of available protein sources as FM substitutes, the effects of tested protein sources on antioxidant capacity and oxidative stress status of aquatic animals should be fully concerned.

Several former literatures have clarified that maintaining healthy morphology and structure of intestinal mucosa are of great importance in nutrient absorption and resistance to pathogenic microorganisms [13, 31]. The VH, VW, and MT are well-applied to evaluate health status of intestinal mucosal structure, and decrease of VH, VW, and MT indicates intestinal mucosal structure damage. In current study, we observed that the FSM75 and FSM100 diets obviously decreased anterior intestinal VH, VW, and MT. The VH, VW, and MT exhibited significant negative linear and quadratic trends with the substitution level of dietary FM with FSM. As a result, these data suggested that replacing excessive dietary FM by FSM was not beneficial to maintain the intestinal health of large yellow croaker. To my knowledge, it was the first study that determined the pernicious influences of excessive dietary FM replacement with FSM on the intestinal health of large yellow croaker.

The intestinal mucosa mainly consisted of a single layer of intestinal epithelial cells, which are sealed with intestinal tight junction proteins (TJPs) [32]. Intestinal TJPs are one of the main components of intestinal mechanical barrier [33]. The disruption of intestinal TJPs leads to the increase of permeability of intestinal mucosa, which further induced the occurrence of intestinal inflammation and intestinal mucosa structural injury [34]. Thus, in the cause of identifying the mechanism of excessive dietary FM replacement with FSM-induced intestinal mucosal structure damage, we determined the influences of dietary FM replaced with FSM on intestinal barrier and inflammatory response. The present study suggested that the transcripts levels of intestinal barrier-related genes (*pcna*, *zo-1*, *zo-2*, and *occludin*) and anti-inflammatory response-related genes (*il-4/13a*, *il-4/13b*, *il-10*, and *tgfβ*) were significantly decreased, and the mRNA abundances of proinflammatory gene *il-6* were markedly increased in FSM50, FSM75, and FSM100 groups. From these results, we obtained that high-dietary FSM inhibited intestinal barrier function and activated inflammatory response in anterior intestine. Secombes et al. [35] argued that the risk of intestinal inflammation was elevated when intestinal tight junction was damaged. Consistent inflammation resulted in cell apoptosis and ultimately triggered tissue injury [36]. Collectively, we supposed that high-dietary FSM-induced intestinal mucosal structure injury was attributed to the disruption of intestinal TJPs-triggered-chronic inflammation.

Recently, accumulating evidence has revealed that intestinal health is not limited to the structural integrity and good physiological functions capacity of intestinal tissue, and the importance of a balanced and healthy microbial community is also highly appreciated [37]. Many studies have suggested a role for gut-resident microbes in modulating host health such as the improvement of intestinal immune function and metabolic remodeling [38]. Moreover, emerging results suggest that composition of gut microbiota is tightly linked to host diet [39]. Hence, it is meaningful for us to determine the influence of dietary FM substitution by FSM on the intestinal microflora of large yellow croaker. In this study, we found that Fusobacteriota, Firmicutes as well as, Proteobacteria and were major bacterial phylum. In line with the previous study, similar results are also found in the large yellow croaker larvae [40]. The results of intestinal microbial community indicated that when substitution level of FM by FSM was over 25%, relative abundances of Proteobacteria phylum were increased, but relative abundances of Firmicutes phylum were decreased. Proteobacteria is considered as a microbial signature of dysbiosis in gut microbiota [41]. From this perspective, we could acquire that high-dietary FM replacement by FSM might induce microflora dysbiosis in intestine of the large yellow croaker. Furthermore, after LEFSe analysis, we found that replaced diets dramatically reduced the relative abundances of genus Marinobacterium, genus Bacillus, and genus Exiguobacterium. Marinobacterium is rich in marine environments and plays important roles in carbon source utilization and polyhydroxyalkanoate metabolism [42]. Bacillus is viewed as a class of intestinal commensal bacteria, and its spore form is universally utilized as a probiotic [43]. Exiguobacterium can survive in wide ranges of salinity, temperature, as well as pH, and make use of the various proteins and polysaccharides [44]. Hence, we speculated that higher relative abundances of these bacteria might contribute to the maintenance of health status of large yellow croaker fed control diet.

In addition to the above-mentioned, the present study also determined the influences of dietary FM replacement with FSM on protein metabolism in the dorsal muscle of large yellow croaker. Compared with control diet, FSM75 and FSM100 diets significantly decreased phosphorylation levels of mTOR (Ser2448), and replaced diets significantly reduced phosphorylation levels of 4E-BP1 (Thr37/46). The mTOR is a critical nutrient-sensing protein that controls multiple aspects of protein synthesis [45]. The phosphorylation of mTOR at the site of Ser2448 efficiently activates mTOR activity and enhances protein synthesis [45]. The mTOR activation modulates two crucial effectors of translation, 4E-BP1 and S6K [46]. mTORC1 phosphorylates 4E-BP1 at multiple sites (Thr37/Thr46 and Ser65/Thr70), causing the dissociation of 4E-BP1 from eIF4E, which permits eIF4F complex assembly and promotes 5′ cap-dependent mRNA translation [47]. Therefore, these results suggested that high-dietary FM substitution by FSM might impair protein deposition in muscle of the large yellow croaker.

## 5. Conclusion

In current study, we determined previously unknown effects of dietary FM replaced by FSM on growth performance, antioxidant capacity, intestinal health and microflora, inflammatory response, and protein metabolism of large yellow croaker. The current study illustrated that 25% dietary FM was able to be substituted by FSM without weakening the growth performance and intestinal health of large yellow croaker. In addition, high level of dietary FM substitution by FSM (over 25%) will ultimately cause adverse effects on large yellow croaker. High inclusion of dietary FSM might cause decreased growth performance by reducing digestive enzymes activity, and induce intestinal mucosal structure injury by disruption of intestinal TJPs-triggered chronic inflammation.

## Figures and Tables

**Table 1:** Formulation and proximate composition of the experimental diets (% dry matter).

Ingredients	Diets				
	FSM0^3^	FSM25	FSM50	FSM75	FSM100
Fish meal^1^	40	30	20	10	0
Fish steak meal^1^	0.00	13.34	26.68	40.03	53.38
Soybean meal^1^	12.00	12.00	12.00	12.00	12.00
Soybean protein concentrate^1^	10.00	10.00	10.00	10.00	10.00
Wheat flour^1^	15.00	15.00	15.00	15.00	15.00
Fish oil^1^	2.62	1.99	1.35	0.71	0.07
Soybean oil^1^	2.62	1.99	1.35	0.71	0.07
Soybean lecithin^1^	1.50	1.50	1.50	1.50	1.50
Ca(H_2_PO_4_)_2_	1.50	1.50	1.50	1.50	1.50
Choline chloride^1^	0.30	0.30	0.30	0.30	0.30
Mineral premix^2^	2.00	2.00	2.00	2.00	2.00
Vitamin premix^2^	3.00	3.00	3.00	3.00	3.00
Cellulose	9.46	7.38	5.32	3.25	1.18
Proximate composition					
Crude protein	44.2	43.95	44.17	44.39	44.20
Crude lipid	9.76	9.31	9.55	9.71	9.67
Moisture	12.26	11.51	12.07	11.82	12.35
Ash	9.83	11.67	13.70	15.42	16.92

^1^These ingredients were purchased from the Fuzhou Haima Feed Co., Ltd., (Fuzhou, China). ^2^The component of vitamin premix and mineral premix were based on the previous study [24]. ^3^FSM, fish steak meal.

**Table 2:** The nutritional composition of fish meal (FM) and fish steak meal (FSM)^1^.

Index	FM^2^	FSM^2^
Moisture (%)	7.64	7.09
Crude protein (%/dry matter)	72.28	54.15
Crude lipid (%/dry matter)	6.68	14.56
Amino acids (%/dry matter)		
Essential amino acids		
Arginine	4.35	3.68
Histidine	2.38	1.85
Isoleucine	3.26	2.35
Leucine	5.50	4.32
Lysine	5.76	4.57
Methionine	2.14	1.23
Phenylalanine	3.05	2.41
Threonine	3.11	2.45
Valine	3.83	2.64
Nonessential amino acids		
Aspartic acid	6.4	4.38
Glutamic acid	10.53	9.76
Serine	2.87	2.05
Proline	2.67	2.49
Glycine	4.30	1.96
Alanine	4.76	2.53
Tyrosine	2.39	1.43
Cysteine	0.58	0.35

^1^Data are means of triplicate. No tryptophan was detected because of acid hydrolysis. ^2^Fish meal (FM) and fish steak meal (FSM) were provided by the Fuzhou Haima Feed Co. Ltd., (Fuzhou, China).

**Table 3:** Primers used for real-time quantitative PCR.

Genes^1^	Forward primer (5′–3′)	Reverse primer (5′–3′)	Accession number
*claudin-11*	ACCTCCGCCATCAAGCA	TGGGACAAAGAGCCACATC	XM_010732201.3
*il-10*	AGTCGGTTACTTTCTGTGGTG	TGTATGACGCAATATGGTCTG	XM_010738826.3
*il-1β*	CAGCTGTTCTCAAGTATGTGGC	GTTGTAAATAGTGGGTGTGTCG	XM_010736551.3
*il4-13a*	TGGTACTGCTGGTCAATCCG	TTTTGCCTTCAGCCAGATGT	KU885454
*il4-13b*	AGTTCTTCTGTCGCGCTGAG	GCTATGTATGTGCGGTTGCTG	KU885453
*il-6*	GCTGTTCTCAAGTATGTGGCG	TGTTGTAAATAGTGGGTGTGTCG	XM_010734753.3
*occludin*	AGGCTACGGCAACAGTTATG	GTGGGTCCACAAAGCAGTAA	XM_010734512.3
*pcna*	AGTTTGCCCGTATCTGCC	CTCTTTGTCTACATTGCTGGTCT	XM_010734227.3
*tgfβ*	AGCAACCACCGTACATCCTG	AGGTATCCCGTTGGCTTGTG	XM_027280465.1
*tnfα*	ACACCTCTCAGCCACAGGAT	CCGTGTCCCACTCCATAGTT	XM_010745990
*zo-1*	TGTCAAGTCCCGCAAAAATG	CAACTTGCCCTTTGACCTCT	XM_010731239.3
*zo-2*	ACCCGACCTGTTTGTTATTG	ATGCCGTGCTTGCTGTC	XM_027276911.1
*β-actin*	GACCTGACAGACTACCTCATG	AGTTGAAGGTGGTCTCGTGGA	GU584189

^1^ *il-10*, Interleukin-10; *il-1β*, interleukin-1*β*; *il4-13a*, *interleukin-4-13a*; *il4-13b*, *interleukin-4-13b; il-6*, interleukin-6; *pcna*, proliferating cell nuclear antigen; *tgfβ*, transforming growth factor *β*; *tnfα*: tumor necrosis factor *α*; *zo-1*, zonula occludens-1; nd zo-2, zonula occludens-2.

**Table 4:** Growth performance and feed utilization of large yellow croaker fed the different experimental diets (mean ± SD, *n* = 4)^1^.

Index^2^	Diets					*P* value		
	FSM0	FSM25	FSM50	FSM75	FSM100	ANOVA	Linear	Quadratic
FBW (g)	82.63 ± 7.82^a^	82.11 ± 7.22^a^	72.03 ± 9.19^ab^	72.92 ± 11.14^ab^	64.46 ± 6.05^b^	0.042	0.002	0.010
WG (%)	330.77 ± 30.77^a^	306.76 ± 13.41^ab^	279.81 ± 21.76^bc^	267.93 ± 14.83^cd^	232.85 ± 30.34^d^	<0.001	<0.001	<0.001
SR (%)	66.25 ± 2.85	65.00 ± 4.90	65.42 ± 6.72	63.75 ± 6.72	62.08 ± 6.58	0.861	0.266	0.527
SGR (%/day)	2.60 ± 0.13^a^	2.50 ± 0.06^ab^	2.38 ± 0.10^bc^	2.33 ± 0.07^c^	2.14 ± 0.16^d^	<0.001	<0.001	<0.001
FCR	1.53 ± 0.07^c^	1.64 ± 0.14^bc^	1.81 ± 0.24^b^	2.05 ± 0.11^a^	2.06 ± 0.09^a^	<0.001	<0.001	<0.001
PER	1.48 ± 0.06^a^	1.40 ± 0.12^ab^	1.27 ± 0.18^b^	1.10 ± 0.06^c^	1.10 ± 0.05^c^	<0.001	<0.001	<0.001

^1^Means in the same row sharing the same superscript letter are not significantly different. The significance threshold was *P* < 0.05. ^2^FBW, final body weight; feed conversion rate (FCR) = dry feed fed (g)/wet weight gain (g); FSM, fish steak meal; protein efficiency ratio (PER) = wet weight gain (g)/dry protein fed (g); specific growth rate (SGR, %/day) = (Ln *W*_*t*_ − Ln *W*_0_) × 100/t; survival rate (SR, %) = 100 × (*N*_*t*_/*N*_0_); weight gain (WG, %) = (*W*_*t*_ − *W*_0_) × 100/*W*_0_. *W*_0_ and *W*_*t*_ represent the initial, final body weight, respectively. *N*_0_ and *N*_*t*_ are initial and final number of fish.

**Table 5:** The digestive enzymes activity and oxidative stress-related parameters in the liver of large yellow croaker fed the different experimental diets (mean ± SD, *n* = 4)^1^.

Index^2^	Diets					*P* value		
	FSM0	FSM25	FSM50	FSM75	FSM100	ANOVA	Linear	Quadratic
AMS (U/mg pro)	1.22 ± 0.08^a^	1.20 ± 0.06^ab^	1.10 ± 0.06^b^	0.95 ± 0.05^c^	0.86 ± 0.05^c^	0.023	0.001	0.003
LPS (U/g pro)	50.58 ± 1.81^a^	49.60 ± 3.26^a^	42.46 ± 2.86^b^	39.13 ± 1.64^bc^	34.95 ± 2.63^c^	<0.001	<0.001	<0.001
Trypsin (U/mg pro)	98.80 ± 2.03^a^	96.42 ± 7.40^ab^	88.73 ± 1.27^bc^	85.60 ± 4.44^c^	84.54 ± 3.66^c^	0.007	<0.001	0.001
CAT (U/mg pro)	70.15 ± 1.85^a^	70.61 ± 3.52^a^	65.33 ± 4.87^a^	57.64 ± 4.18^b^	57.25 ± 3.04^b^	0.002	<0.001	<0.001
T-SOD (U/mg pro)	120.36 ± 9.61^a^	77.81 ± 10.28^b b^	71.04 ± 9.98^bc^	65.42 ± 6.54^bc^	59.93 ± 4.58^c^	<0.001	<0.001	<0.001
T-AOC (U/mg pro)	0.50 ± 0.07^a^	0.45 ± 0.05^ab^	0.40 ± 0.02^b^	0.32 ± 0.03^c^	0.30 ± 0.03^c^	0.001	<0.001	<0.001
MDA (nmol/mg pro)	3.88 ± 0.30^b^	4.02 ± 0.39^b^	4.18 ± 0.13^b^	5.47 ± 0.41^a^	6.07 ± 0.36^a^	<0.001	<0.001	<0.001

^1^Means in the same row sharing the same superscript letter are not significantly different. The significance threshold was *P* < 0.05. ^2^AMS, *α*-amylase; CAT, catalase; FSM, fish steak meal; LPS, lipase; MDA, malonyldialdehyde; T-AOC, total antioxidant capacity; and T-SOD, total superoxide dismutase.

**Table 6:** The effects of fish meal replaced by fish steak meal on alpha diversity index of intestinal microbiota of large yellow croaker fed the different experimental diets (mean ± SD, *n* = 3).

Index	FSM0^1^	FSM25	FSM50	FSM75	FSM100
Sobs	69.67 ± 13.80	48.67 ± 15.95	42.67 ± 2.52	45.33 ± 11.68	56.00 ± 9.54
Shannon	1.43 ± 0.65	1.40 ± 0.31	1.45 ± 0.37	1.34 ± 0.53	1.11 ± 0.64
Simpson	0.40 ± 0.29	0.35 ± 0.14	0.32 ± 0.11	0.43 ± 0.25	0.54 ± 0.29
Ace	71.05 ± 12.33	74.11 ± 1.27	49.39 ± 7.57	47.27 ± 10.40	57.90 ± 10.55
Chao	70.69 ± 12.12	59.47 ± 10.61	48.48 ± 4.66	46.12 ± 11.29	57.43 ± 10.57
Coverage	0.9999 ± 0.0001	0.9997 ± 0.0001	0.9998 ± 0.0001	0.9999 ± 0.0000	0.9999 ± 0.0001

^1^FSM, fish steak meal.

## Data Availability

The data that support the findings of this study are available from the corresponding author upon request.

## Supplementary Materials

Supplementary Materials Figure S1: the Venn diagram for intestinal microflora of large yellow croaker on OTU level. Figure S2: relative abundance of microbial community in intestine of large yellow croaker on phylum level. Figure S3: LEfSe analysis identified the most differentially abundant taxons among the five groups.

## References

[1] Galkanda-Arachchige H. S. C., Wilson A. E., Davis D. A. (2020). Success of fishmeal replacement through poultry by-product meal in aquaculture feed formulations: a meta-analysis. *Reviews in Aquaculture*.

[2] FAO (2020). *The State of World Fisheries and Aquaculture*.

[3] Liu T., Han T., Wang J. (2021). Effects of replacing fish meal with soybean meal on growth performance, feed utilization and physiological status of juvenile redlip mullet *Liza haematocheila*. *Aquaculture Reports*.

[4] Wu Y. B., Wang Y., Ren X., Huang D., Si G. R. E., Chen J. M. (2021). Replacement of fish meal with gamma-ray irradiated soybean meal in the diets of largemouth bass *Micropterus salmoides*. *Aquaculture Nutrition*.

[5] Yang Y., Xie S., Lei W., Zhu X., Yang Y. (2004). Effect of replacement of fish meal by meat and bone meal and poultry by-product meal in diets on the growth and immune response of *Macrobrachium nipponense*. *Fish & Shellfish Immunology*.

[6] Toppe J., Aksnes A., Hope B., Albrektsen S. (2006). Inclusion of fish bone and crab by-products in diets for Atlantic cod, *Gadus morhua*. *Aquaculture*.

[7] Lee K.-J., Powell M. S., Barrows F. T., Smiley S., Bechtel P., Hardy R. W. (2010). Evaluation of supplemental fish bone meal made from Alaska seafood processing byproducts and dicalcium phosphate in plant protein based diets for rainbow trout *Oncorhynchus mykiss*. *Aquaculture*.

[8] Kim H. S., Jung W.-G., Myung S. H., Cho S. H., Kim D. S. (2014). Substitution effects of fishmeal with tuna byproduct meal in the diet on growth, body composition, plasma chemistry and amino acid profiles of juvenile olive flounder *Paralichthys olivaceus*. *Aquaculture*.

[9] Xie M. X., Xie Y. D., Li Y. (2021). The effects of fish meal replacement with ultra-micro ground mixed plant proteins (uPP) in practical diet on growth, gut and liver health of common carp (*Cyprinus carpio*). *Reports, Development*.

[10] Chui A., Zhang Q., Dai Q., Shi S. H. (2020). Oxidative stress regulates progenitor behavior and cortical neurogenesis. *Development*.

[11] Lauridsen C. (2019). From oxidative stress to inflammation: redox balance and immune system. *Poultry Science*.

[12] Rashid C. S., Bansal A., Simmons R. A. (2018). Oxidative stress, intrauterine growth restriction, and developmental programming of type 2 diabetes. *Physiology*.

[13] Saleh N. E. (2020). Assessment of sesame meal as a soybean meal replacement in European sea bass (*Dicentrarchus labrax*) diets based on aspects of growth, amino acid profiles, haematology, intestinal and hepatic integrity and macroelement contents. *Fish Physiology and Biochemistry*.

[14] Zhou C., Lin H., Huang Z., Wang J., Wang Y., Yu W. (2020). Effects of dietary leucine levels on intestinal antioxidant status and immune response for juvenile golden pompano *Trachinotus ovatus* involved in Nrf2 and NF-*κ*B signaling pathway. *Fish Shellfish Immunology*.

[15] Lynch S. V., Pedersen O. (2016). The human intestinal microbiome in health and disease. *The New England Journal of Medicine*.

[16] Cai W., Fu L., Liu C. (2023). Dietary ribose supplementation improves flesh quality through purine metabolism in gibel carp (*Carassius auratus gibelio*). *Animal Nutrition*.

[17] Fishery Bureau, Ministry of Agriculture and Rural Affairs (2021). *China Fishery Statistical Yearbook*.

[18] Xu J. M., Sheng Z. Y., Chen N. S., Xie R. T., Zhang H. T., Li S. L. (2022). Effect of dietary fish meal replacement with spray dried chicken plasma on growth, feed utilization and antioxidant capacity of largemouth bass (*Micropterus salmoides*). *Aquacultue Reports*.

[19] Zhang D.-G., Zhao T., Xu X.-J., Lv W.-H., Luo Z. (2021). Dietary marginal and excess selenium increased triglycerides deposition, induced endoplasmic reticulum stress and differentially influenced selenoproteins expression in the anterior and middle intestines of yellow catfish *Pelteobagrus fulvidraco*. *Antioxidants*.

[20] Yao C., Huang W., Liu Y. (2022). Effects of different marine-based lipid sources on growth performance, activities of digestive enzyme, antioxidant responses, and lipid metabolism of large yellow croaker *Larimichthys crocea* larvae. *Aquaculture Nutrition*.

[21] Zhang D. G., Cheng J., Tai Z. P., Luo Z. (2019). Identification of five genes in endoplasmic reticulum (ER) stress-apoptosis pathways in yellow catfish *Pelteobagrus fulvidraco* and their transcriptional responses to dietary lipid level. *Fish Physiology and Biochemistry, Reports*.

[22] Wang X. X., Luo H. J., Zheng Y. Z. (2023). Effects of poultry by-product meal replacing fish meal on growth performance, feed utilization, intestinal morphology and microbiota communities in juvenile large yellow croaker (*Larimichthys crocea*). *Aquaculture Reports*.

[23] Chen G. H., Song C. C., Zhao T. (2022). Mitochondria-dependent oxidative stress mediates ZnO nanoparticle (ZnO NP)-induced mitophagy and lipotoxicity in freshwater teleost fish. *Environmental Science & Technology*.

[24] Uyan O., Koshio S., Teshima S.-I. (2006). Growth and phosphorus loading by partially replacing fishmeal with tuna muscle by-product powder in the diet of juvenile Japanese flounder, *Paralichthys olivaceus*. *Aquaculture*.

[25] Zhang C., Rahimnejad S., Wang Y.-R. (2018). Substituting fish meal with soybean meal in diets for Japanese seabass *Lateolabrax japonicus*: effects on growth, digestive enzymes activity, gut histology, and expression of gut inflammatory and transporter genes. *Aquaculture*.

[26] Barlaya G., Kumar B. S. A., Huchchappa R. C., Basumatary P., Kannur H. (2021). Effect of fish meal replacement with toasted guar meal on growth, food conversion, digestive enzyme activity and final carcass composition of rohu Labeo rohita. *Aquaculture Research*.

[27] Yu X., Wu Z., Guo J. (2022). Replacement of dietary fish meal by soybean meal on growth performance, immunity, anti-oxidative capacity and mTOR pathways in juvenile abalone *Haliotis discus hannai* Ino. *Aquaculture*.

[28] Lin Y., Miao L.-H., Pan W.-J. (2018). Effect of nitrite exposure on the antioxidant enzymes and glutathione system in the liver of bighead carp, *Aristichthys nobilis*. *Fish & Shellfish Immunology*.

[29] Song C. C., Pantopoulos K., Chen G. H. (2022). Iron increases lipid deposition via oxidative stress-mediated mitochondrial dysfunction and the HIF1*α*–PPAR*γ* pathway. *Cellular and Molecular Life Sciences*.

[30] Lv W.-H., Zhao T., Pantopoulos K. (2022). Manganese-induced oxidative stress contributes to intestinal lipid deposition *via* the deacetylation of PPAR3B3; at K339 by SIRT1. *Antioxidants & Redox Signaling*.

[31] Akbari P., Braber S., Varasteh S., Alizadeh A., Garssen J., Fink-Gremmels J. (2017). The intestinal barrier as an emerging target in the toxicological assessment of mycotoxins. *Archives of Toxicology*.

[32] Zuhl M., Schneider S., Lanphere K., Conn C., Dokladny K., Moseley P. (2014). Exercise regulation of intestinal tight junction proteins. *British Journal of Sports Medicine*.

[33] Martínez C., González-Castro A., Vicario M., Santos J. (2012). Cellular and molecular basis of intestinal barrier dysfunction in the irritable bowel syndrome. *Gut and Liver*.

[34] DeMeo M. T., Mutlu E. A., Keshavarzian A., Tobin M. C. (2002). Intestinal permeation and gastrointestinal disease. *Journal of Clinical Gastroenterology*.

[35] Secombes C. J., Wang T., Hong S. (2001). Cytokines and innate immunity of fish. *Developmental and Comparative Immunology*.

[36] Gong Y., Lu Q., Liu Y. (2022). Dietary berberine alleviates high carbohydrate diet-induced intestinal damages and improves lipid metabolism in largemouth bass (*Micropterus salmoides*). *Frontiers in Nutrition*.

[37] Girbovan A., Sur G., Samasca G., Lupan I. (2017). Dysbiosis a risk factor for celiac disease. *Medical Microbiology and Immunology*.

[38] Saeedi B. J., Liu K. H., Owens J. A. (2020). Gut-resident lactobacilli activate hepatic Nrf2 and protect against oxidative liver injury. *Cell Metabolism*.

[39] Tang W. H. W., Li D. Y., Hazen S. L. (2019). Dietary metabolism, the gut microbiome, and heart failure. *Nature Reviews Cardiology*.

[40] Lv H. R., Liu Y. L., Li H. D. (2021). Modulation of antioxidant enzymes, heat shock protein, and intestinal microbiota of large yellow croaker (*Larimichthys crocea*) under acute cold stress. *Frontiers in Marine Science*.

[41] Shin N.-R., Whon T. W., Bae J.-W. (2015). Proteobacteria: microbial signature of dysbiosis in gut microbiota. *Trends in Biotechnology*.

[42] Wang M., Xi W., Li Z. (2020). Analysis of the genome sequencing data of the marinobacterium genus. *Chinese journal of biotechnology*.

[43] Cutting S. M. (2011). Bacillus probiotics. *Food Microbiology*.

[44] Zhang D., Zhu Z., Li Y., Li X., Guan Z., Zheng J. (2021). Comparative genomics of exiguobacterium reveals what makes a cosmopolitan bacterium. *mSystems*.

[45] Szwed A., Kim E., Jacinto E. (2021). Regulation and metabolic functions of mTORC1 and mTORC2. *Physiological Reviews*.

[46] Guertin D. A., Sabatini D. M. (2007). Defining the role of mTOR in cancer. *Cancer Cell*.

[47] Battaglioni S., Benjamin D., Wälchli M., Maier T., Hall M. N. (2022). mTOR substrate phosphorylation in growth control. *Cell*.

