# Supplementary material for: Effects of Dietary Fish Meal Replaced by Fish Steak Meal on Growth Performance, Antioxidant Capacity, Intestinal Health and Microflora, Inflammatory Response, and Protein Metabolism of Large Yellow Croaker Larimichthys crocea
Source: Aquac Nutr. 2023 Dec 20;2023:2733234. doi: 10.1155/2023/2733234 (PMC10752682; doi:10.1155/2023/2733234)
Supplement: Supplementary Materials — Figure S1: the Venn diagram for intestinal microflora of large yellow croaker on OTU level. Figure S2: relative abundance of microbial community in intestine of large yellow croaker on phylum level. Figure S3: LEfSe analysis identified the most differentially abundant taxons among the five groups. [file 2733234.f1.pdf]

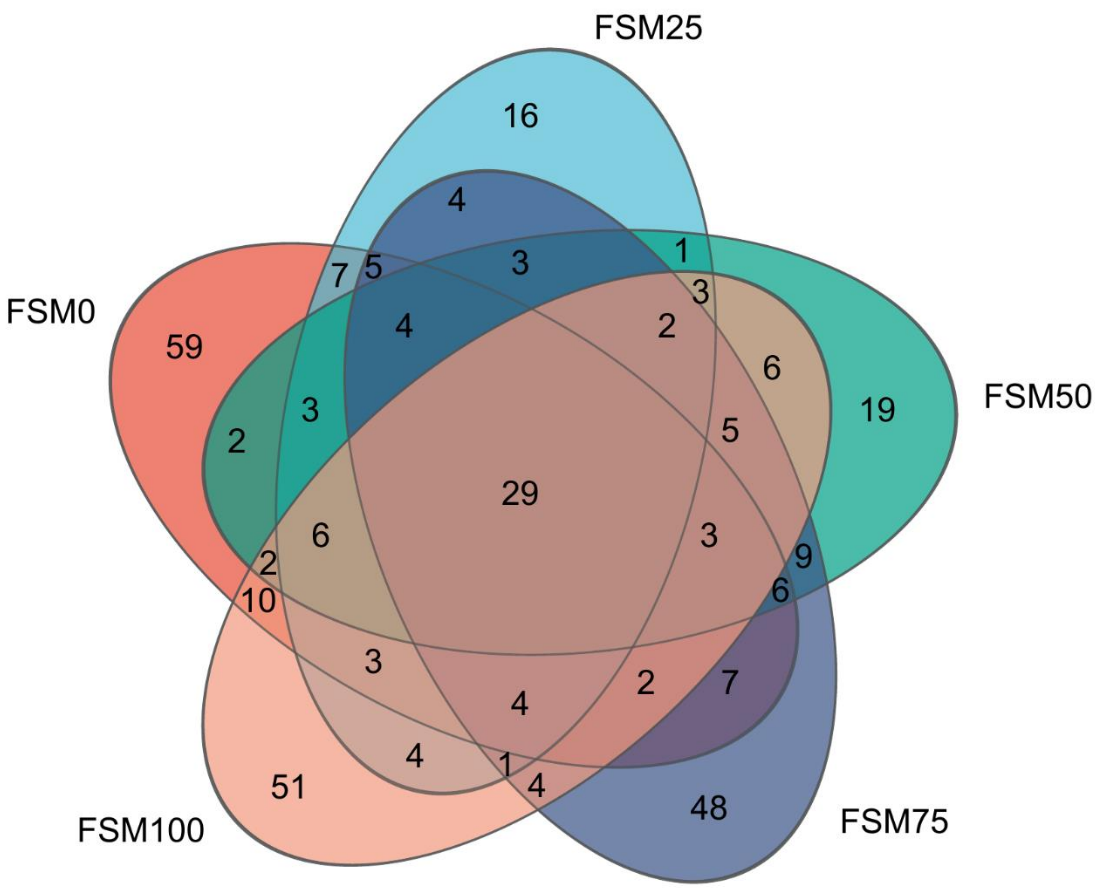

Supplementary Figure 1. The Venn diagram for intestinal microflora of large yellow croaker on OTU level. FSM, fish steak meal.

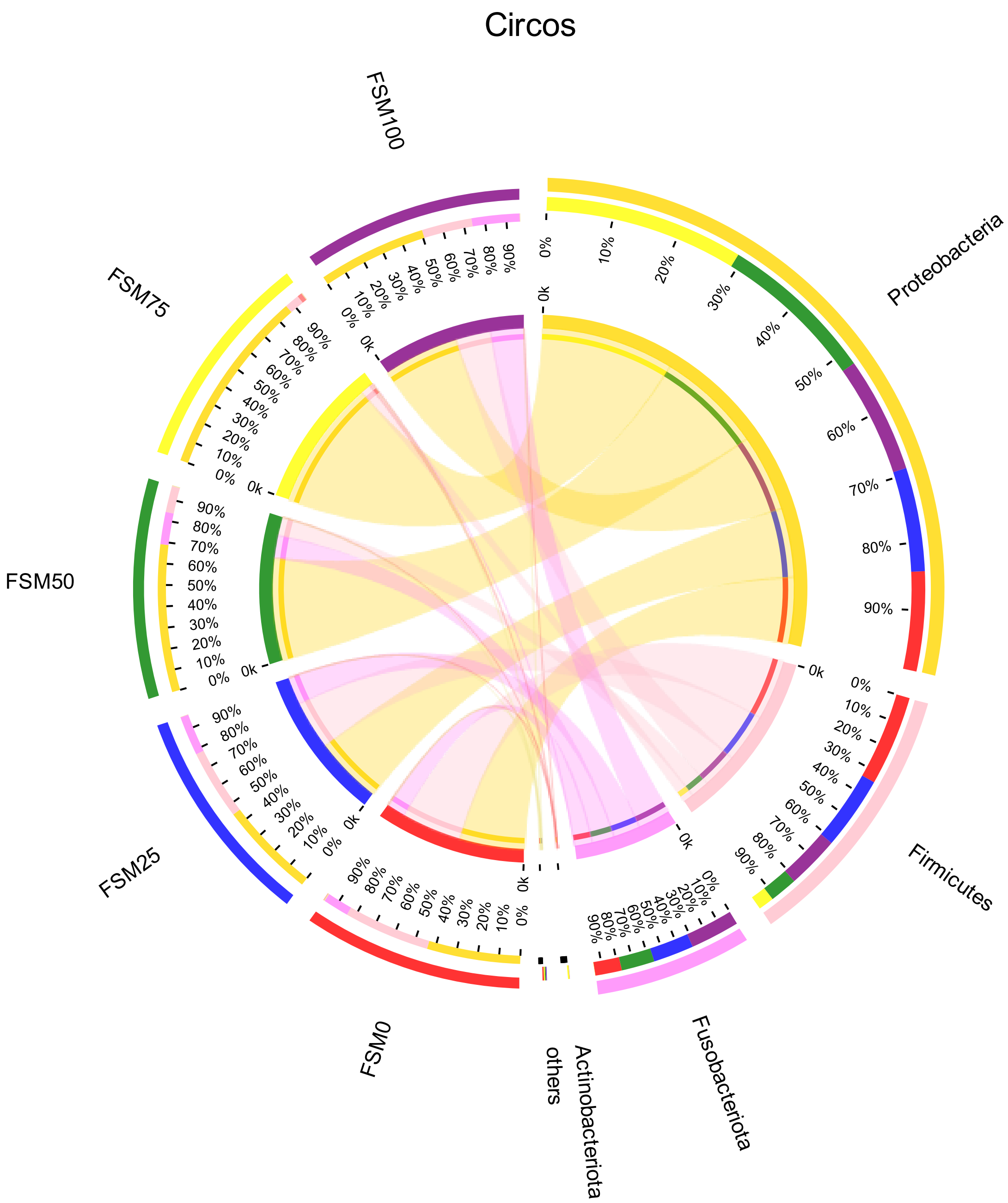

Supplementary Figure 2. Relative abundance of microbial community in intestine of large yellow croaker on phylum level. FSM, fish steak meal.

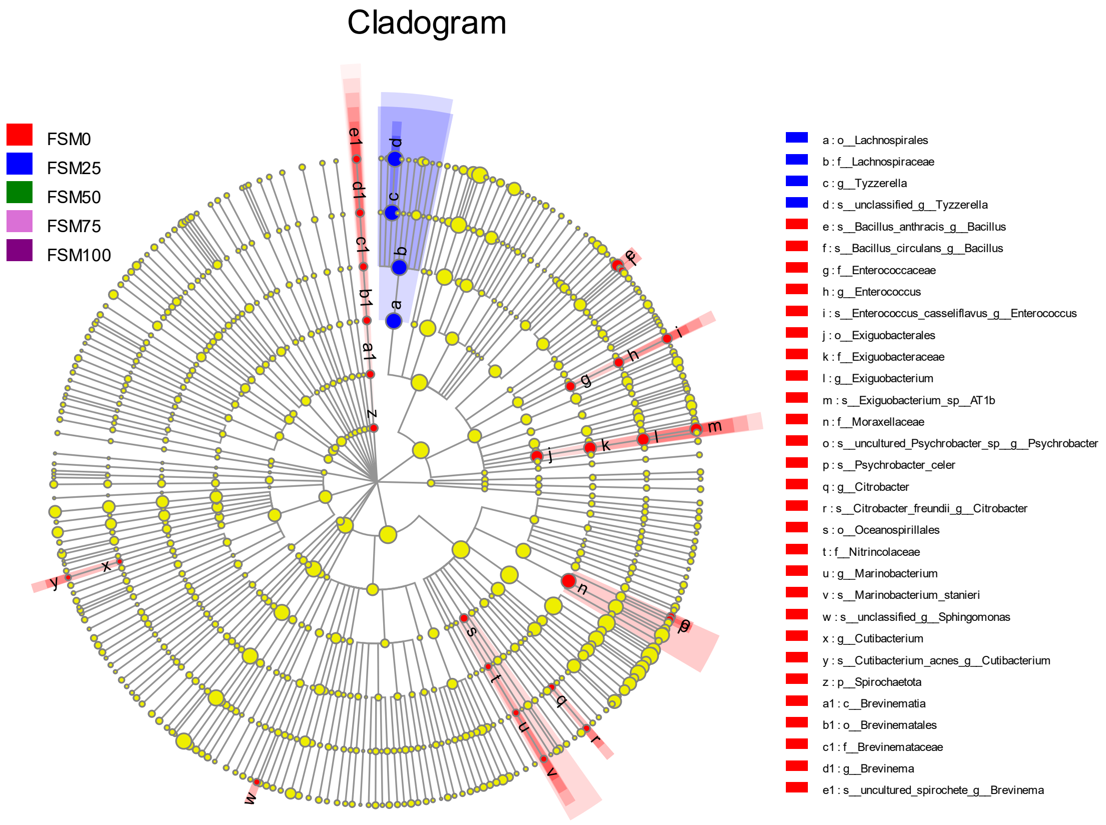

Supplementary Figure 3. LEfSe analysis identified the most differentially abundant taxons among the five groups (n = 3/group). FSM, fish steak meal.
